# Active solution of homography for pavement crack recovery with four laser lines

**DOI:** 10.1038/s41598-018-25572-9

**Published:** 2018-05-08

**Authors:** Guan Xu, Fang Chen, Guangwei Wu, Xiaotao Li

**Affiliations:** 1 Transportation College, Nanling Campus, Jilin University, Renmin Str. 5988#, Changchun, China; 2 School of Mechanical Science and Engineering, Nanling Campus, Jilin University, Renmin Str. 5988#, Changchun, China

## Abstract

An active solution method of the homography, which is derived from four laser lines, is proposed to recover the pavement cracks captured by the camera to the real-dimension cracks in the pavement plane. The measurement system, including a camera and four laser projectors, captures the projection laser points on the 2D reference in different positions. The projection laser points are reconstructed in the camera coordinate system. Then, the laser lines are initialized and optimized by the projection laser points. Moreover, the plane-indicated Plücker matrices of the optimized laser lines are employed to model the laser projection points of the laser lines on the pavement. The image-pavement homography is actively determined by the solutions of the perpendicular feet of the projection laser points. The pavement cracks are recovered by the active solution of homography in the experiments. The recovery accuracy of the active solution method is verified by the 2D dimension-known reference. The test case with the measurement distance of 700 mm and the relative angle of 8° achieves the smallest recovery error of 0.78 mm in the experimental investigations, which indicates the application potentials in the vision-based pavement inspection.

## Introduction

Vision-based inspection^1–4^ is broadly applied in the fields of the mechanical part manufacture^5,6^, the dimension measurement^7,8^, the diagnostic equipment^9–11^ and Fourier profilometry^12–14^, etc. The crack on the pavement is one of the most important inspection objects in the vision-based inspection. Although the road surfaces with the extreme roughness do not evidently cause the serious traffic accidents due to the cautiousness of the drivers^15^, the previous studies indicate that a significant decrease in the road capacity of about 30% is attributable to the road pavement distress^16^. Moreover, the two-lane-road capacity is augmented by 10–15% according to a perfect driving surface^17^. Thus, the quantitive detection and evaluation of the crack are beneficial to extend the lifetime of the pavement^18^ and enhance the driving quality as well as the traffic safety^19^.

The inspections of the pavement cracks include three kinds of methods. The first kind of methods is the measurement on the basis of the linear array camera^20^. Mraz A.^21^ constructs a pavement imaging system with the line array camera at a preset height and a computer. According to different light conditions, an additional lighting system is designed for the inspection system. The accuracy of the pavement image system is evaluated on different lighting conditions. The detection system named the automated pavement distress survey (APDS) is designed by Yao M.^22^ to achieve the automatic inspection of pavement cracks. The developed system consists of two line-scan cameras. The exposure settings of the two cameras are different to deal with different lighting conditions. The calibration method for the line-scan cameras and the image fusion approach are introduced in the study. The linear array camera has only one line of photosensitive elements. Therefore, it takes the advantages of the high resolution and frequency. However, in the scanning applications, the linear array camera is fixed on the moving vehicle. As the speed of the vehicle and the lateral sliding is difficult to measure accurately, the information combination of the pavement cracks is the problem to solve. The second kind of methods is provided by the laser scanner^23^. Li Q.^24^ presents a pavement generation method with the triangulation of structured light. The method consists of filtering, edge detection, spline interpolation, and laser stripe location. The pavement surface is derived from the laser stripe on the pavement. The laser scanner reconstructs the pavement cracks by 3D information. Li L.^25^ proposes a bounding box-based technique in order to separate the captured cracks to appropriate types. The cracks are recognized by a seed fusion method and a pavement generation system. The bounding box is determined by road marks and wheel paths, by which the cracks are classified and measured. Li W.^26^ outlines a detection method for the pavement cracks, on the basis of the empirical mode decomposition (EMD). The region-grow method and morphology are performed on the binary crack images. A deep-learning network, instead of the convolutional neural network, is employed by Zhang A.^27^ for the pavement crack detection. The method eliminates the pooling layers to simple the outputs of former layers. The laser-scanner-based approaches reconstruct the pavement cracks by 3D information. Nevertheless, the laser scanner on the vehicle tends to be influenced by the weather and cannot contribute the color information of the measured object. In addition, the laser scanner is the much more expensive than the cameras for the normal applications. The third kind of methods refers to capture the image by the planar array camera^28^. Tsai Y.^29^ evaluates the image segmentation methods, including the statistical thresholding, the edge detection, the multiscale wavelets, the iterative clipping and the dynamic optimization, for the pavement crack sealing. The pavement images with diverse lighting conditions and cracks are provided to test the method performances. The planar array camera is the measurement technology that is widely used in the most vision-based cases. The planar array camera directly captures the 2D image in the test. Hence, it is an effective and economical way for the situations requiring the moderate solution. Nevertheless, in the pavement crack measurement, the images captured from the planar array camera are measured by image pixels. The pavement cracks are dimensioned by millimeters. Thus, the cracks in the image plane should be transformed to the real cracks on the pavement plane. The pavement plane can be generated from a 2D dimension-known reference on the pavement and the Zhang’s method. The bridge from the image plane to the pavement plane is represented by a 2D-2D homography. However, the homography is not a constant matrix due to the relative motion between the camera fixed on the vehicle and the pavement. Thus, an active solution approach is proposed to contribute the homography from the image to the pavement. There are 8 freedoms of the 2D-2D homography without regard to the global freedom. Moreover, a pair of corresponding points determines 2 freedoms in the homography. Therefore, 4 laser lines, which aim to achieve the minimum number of the laser projection points on the pavement, are chosen in the active solution method.

The rest paper consists of three parts. Section 2 constructs the geometrical model of the active solution of the homography. The laser points of the laser lines are derived from the projections on the 2D reference in different positions. Then, the 3D laser lines are initialized and optimized by the 3D laser points in the camera coordinate system. Finally, the laser lines are projected to the pavement. The homography is determined by the laser projections on the pavement and the related image points. Section 3 performs the experiments to recover the pavement cracks with the active solution of homography. The recovery accuracy is also estimated in the experiments. Section 4 provides the conclusion.

## Geometrical Model of Homography

**Figure 1:**
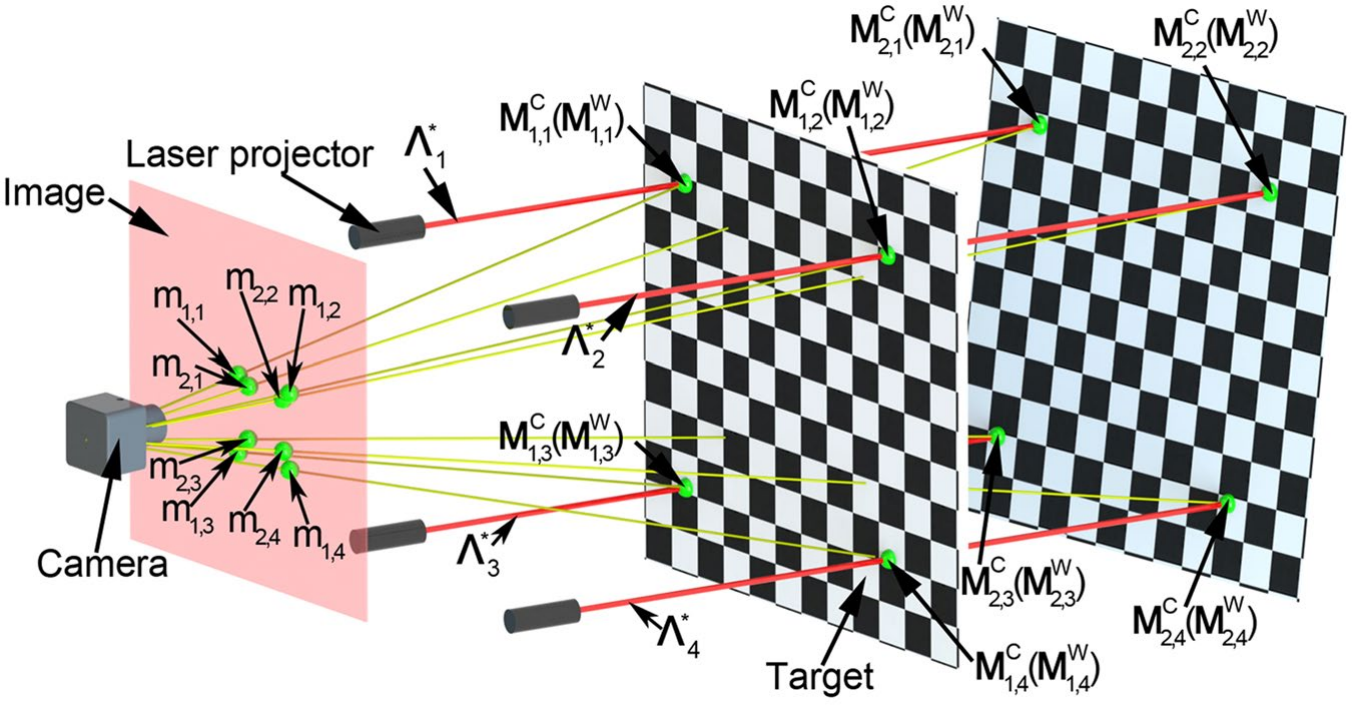
Calibration method of the laser lines with the intersection points in the camera coordinate system. The measurement system consists of four laser projectors and a camera. The positions of the projectors are fixed relative to the camera. A planar target is employed as the reference of the calibrations for the camera and laser lines.

The measurement system, as illustrated in Fig. 1, consists of four laser projectors and a camera. The positions of the projectors are fixed relative to the camera. A planar target is employed as the reference of the calibrations for the camera and laser lines. The world coordinate system, the camera coordinate system and the image coordinate system are attached on the target, camera and image, respectively. Here, the camera coordinate system is considered as the global coordinate system.

The target is moved to different positions in the view field of the camera. Therefore, the laser lines intersect the target at the laser points on the target. The laser point in the world coordinate system is projected to the image and recovered by^30^

1 $${\hat{{\bf{M}}}}_{i,j}^{{\rm{W}}}=[{{\bf{r}}}_{i,1},{{\bf{r}}}_{i,2},{{\bf{t}}}_{i}{]}^{-1}{{\rm{K}}}^{-1}{{\bf{m}}}_{i,j}^{{\rm{W}}}$$

where ${\hat{{\bf{M}}}}_{i,j}^{{\rm{W}}}$ is the homogeneous coordinates of the *x*, *y* coordinates of the laser point in the world coordinate system. As the *z* coordinate is zero on the target plane, the laser point ${{\bf{M}}}_{i,j}^{{\rm{W}}}$ can be derived from ${\hat{{\bf{M}}}}_{i,j}^{{\rm{W}}}$. *i* = 1, 2, …, *n* is the number of the positions of the target. *j* = 1, 2, 3, 4 is the number of the laser lines. K is the intrinsic parameter matrix of the camera. R_*i*_ = [**r**_*i*,1_, **r**_*i*,2_, **r**_*i*,3_] and **t**_*i*_ are the rotation matrix and translation vector from the world coordinate system to the global coordinate system. Zhang’s method^30^ is chosen to calibrate K, R_*i*_**t**_*i*_. ${{\bf{m}}}_{i,j}^{{\rm{W}}}$ is the laser point in the image coordinate system.

In order to represent the laser points in the global coordinate system, the laser point is transformed to^31^

2 $${{\bf{M}}}_{i,j}^{{\rm{C}}}{=[{\rm{R}}}_{i},{{\bf{t}}}_{i}]{{\bf{M}}}_{i,j}^{{\rm{W}}}$$

where ${{\bf{M}}}_{i,j}^{{\rm{C}}}={[{x}_{i,j}^{{\rm{C}}},{y}_{i,j}^{{\rm{C}}},{z}_{i,j}^{{\rm{C}}},1]}^{{\rm{T}}}$ is the laser point in the camera coordinate system.

In the set of the laser points in the camera coordinate system, two laser points ${{\bf{M}}}_{1,j}^{{\rm{C}}}$, ${{\bf{M}}}_{2,j}^{{\rm{C}}}$ far away from each other in the *j*-th laser line are chosen to initially define the laser line by

3 $$\frac{{x}_{i,j}^{{\rm{C}}}-{x}_{1,j}^{{\rm{C}}}}{{x}_{2,j}^{{\rm{C}}}-{x}_{1,j}^{{\rm{C}}}}=\frac{{y}_{i,j}^{{\rm{C}}}-{y}_{1,j}^{{\rm{C}}}}{{y}_{2,j}^{{\rm{C}}}-{y}_{1,j}^{{\rm{C}}}}=\frac{{z}_{i,j}^{{\rm{C}}}-{z}_{1,j}^{{\rm{C}}}}{{z}_{2,j}^{{\rm{C}}}-{z}_{1,j}^{{\rm{C}}}}.$$

Then the parameterized displacements from the other laser points in the set to the laser line are adopted to refine the laser line. The optimization function derived from the sum of the displacements is given by

4 $$f({x}_{1,j}^{{\rm{C}}},{y}_{1,j}^{{\rm{C}}},{z}_{1,j}^{{\rm{C}}},{x}_{2,j}^{{\rm{C}}},{y}_{2,j}^{{\rm{C}}},{z}_{2,j}^{{\rm{C}}})=\sum _{i=1}^{n}\frac{\parallel {({x}_{i,j}^{{\rm{C}}}-{x}_{1,j}^{{\rm{C}}},{y}_{i,j}^{{\rm{C}}}-{y}_{1,j}^{{\rm{C}}},{z}_{i,j}^{{\rm{C}}}-{z}_{1,j}^{{\rm{C}}})}^{{\rm{T}}}\times {({x}_{2,j}^{{\rm{C}}}-{x}_{1,j}^{{\rm{C}}},{y}_{2,j}^{{\rm{C}}}-{y}_{1,j}^{{\rm{C}}},{z}_{2,j}^{{\rm{C}}}-{z}_{1,j}^{{\rm{C}}})}^{{\rm{T}}}\parallel }{\parallel {({x}_{2,j}^{{\rm{C}}}-{x}_{1,j}^{{\rm{C}}},{y}_{2,j}^{{\rm{C}}}-{y}_{1,j}^{{\rm{C}}},{z}_{2,j}^{{\rm{C}}}-{z}_{1,j}^{{\rm{C}}})}^{{\rm{T}}}\parallel }$$

where ${x}_{1,j}^{{\rm{C}}},{y}_{1,j}^{{\rm{C}}},{z}_{1,j}^{{\rm{C}}},{x}_{2,j}^{{\rm{C}}},{y}_{2,j}^{{\rm{C}}},{z}_{2,j}^{{\rm{C}}}$ are the unknown parameters that define the laser line. The parameters are initialized by the results of Eq. (3) and identical to the arguments related to the minimization of the function.

**Figure 2:**
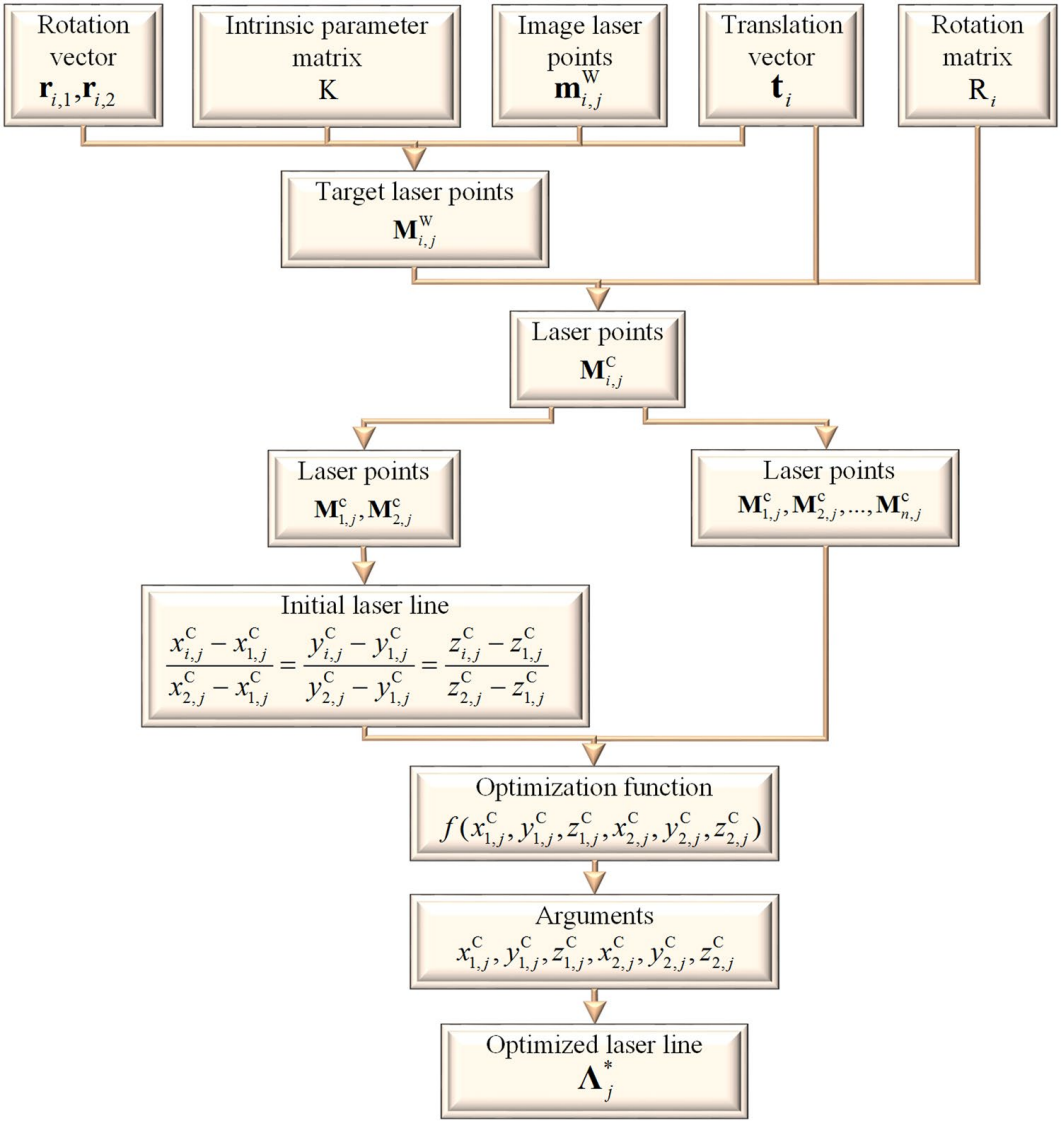
The solution process of the optimized laser line that is derived from the intersection laser points.

Due to Eq. (4) and the Graßmann-Plücker relation^32^, the optimized laser line can be represented by the Plücker matrix

5 $${{\rm{\Lambda }}}_{j}^{\ast }=[\begin{array}{cccc}0 & {y}_{2,j}^{{\rm{C}}}{\,z}_{1,j}^{{\rm{C}}} & -{({y}_{2,j}^{{\rm{C}}})}^{2} & {y}_{2,j}^{{\rm{C}}}({z}_{1,j}^{{\rm{C}}}{\,y}_{2,j}^{{\rm{C}}}-{y}_{1,j}^{{\rm{C}}}{\,z}_{1,j}^{{\rm{C}}})\\ -{y}_{2,j}^{{\rm{C}}}{\,z}_{1,j}^{{\rm{C}}} & 0 & -{x}_{2,j}^{{\rm{C}}}{\,y}_{2,j}^{{\rm{C}}} & {x}_{1,j}^{{\rm{C}}}{\,y}_{2,j}^{{\rm{C}}}{\,z}_{2,j}^{{\rm{C}}}-{z}_{1,j}^{{\rm{C}}}{\,x}_{2,j}^{{\rm{C}}}{\,y}_{2,j}^{{\rm{C}}}\\ {({y}_{2,j}^{{\rm{C}}})}^{2} & -{x}_{2,j}^{{\rm{C}}}{\,y}_{2,j}^{{\rm{C}}} & 0 & {y}_{2,j}^{{\rm{C}}}({y}_{1,j}^{{\rm{C}}}{\,x}_{2,j}^{{\rm{C}}}-{x}_{1,j}^{{\rm{C}}}{\,y}_{2,j}^{{\rm{C}}})\\ {y}_{2,j}^{{\rm{C}}}({y}_{1,j}^{{\rm{C}}}{\,z}_{1,j}^{{\rm{C}}}-{z}_{1,j}^{{\rm{C}}}{\,y}_{2,j}^{{\rm{C}}}) & {z}_{1,j}^{{\rm{C}}}{\,x}_{2,j}^{{\rm{C}}}{\,y}_{2,j}^{{\rm{C}}}-{x}_{1,j}^{{\rm{C}}}{\,y}_{2,j}^{{\rm{C}}}{\,z}_{1,j}^{{\rm{C}}} & {y}_{2,j}^{{\rm{C}}}({x}_{1,j}^{{\rm{C}}}{\,y}_{2,j}^{{\rm{C}}}-{y}_{1,j}^{{\rm{C}}}{\,x}_{2,j}^{{\rm{C}}}) & 0\end{array}]$$

where ${{\rm{\Lambda }}}_{j}^{\ast }$ is the plane-indicated Plücker matrix of the optimized laser line. The generation process of the optimized laser line is described in Fig. 2.

**Figure 3:**
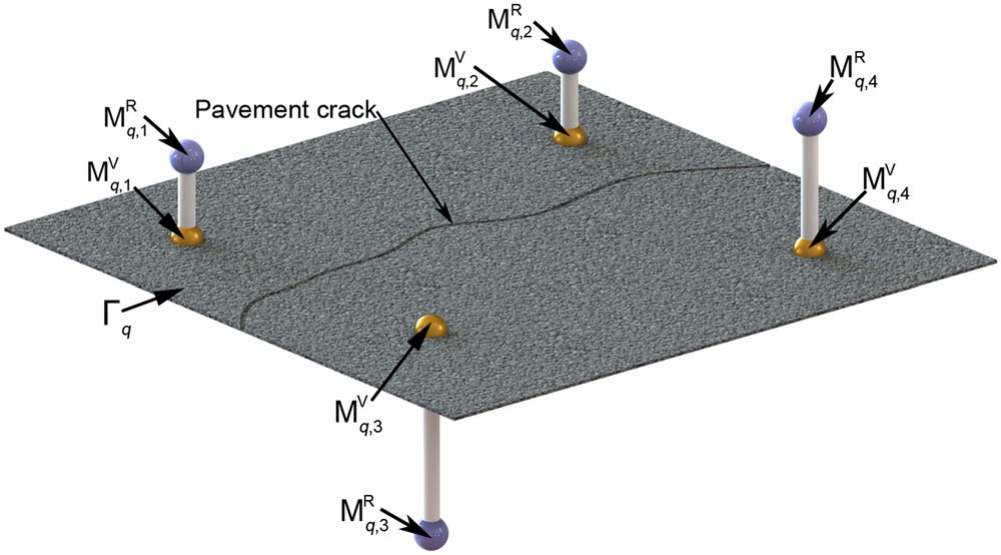
The active solution model of the homography in the camera coordinate system. The laser lines are projected on the pavement. The laser points are derived from the intersections between the laser lines and the pavement plane. All the pavement intersection points are represented in the global coordinate system.

The four laser lines are generated from Eq. (5). The laser projectors and camera are both attached on the vehicle in the pavement test. In Fig. 3, the laser lines are projected on the pavement. The laser points ${{\bf{M}}}_{q,j}^{{\rm{R}}}$ are derived from the intersections between the laser lines and the *q*-th pavement plane, *q* = 1, 2, …, *m*. All the pavement intersection points are represented in the global coordinate system.

The intersection point is on the laser line ${{\rm{\Lambda }}}_{j}^{\ast }$ and satisfies^31^

6 $${{\rm{\Lambda }}}_{j}^{\ast }{{\bf{M}}}_{q,j}^{{\rm{R}}}={{\bf{0}}}_{4\times 1}.$$

The laser point on the pavement also obeys the projection relationship of^33^

7 $${\rm{K}}{{\bf{M}}}_{q,j}^{{\rm{R}}}={s}_{q}{{\bf{m}}}_{q,j}^{{\rm{R}}}$$

where ${{\bf{m}}}_{q,j}^{{\rm{R}}}$ is the image mapping of the laser point on the pavement, *s*_*q*_ is the scale factor.

The laser point on the pavement is generated from Eqs (6) and (7). The laser point obeys the condition of the laser point on the pavement plane^31^. However, in view of the practical non-coplanarity of the four laser points on the pavement, the integrated pavement plane is determined by the four laser points on the pavement as

8 $${[{{\bf{M}}}_{q,1}^{{\rm{R}}},{{\bf{M}}}_{q,2}^{{\rm{R}}},{{\bf{M}}}_{q,3}^{{\rm{R}}},{{\bf{M}}}_{q,4}^{{\rm{R}}}]}^{{\rm{T}}}{{\boldsymbol{\Gamma }}}_{q}={{\bf{0}}}_{4\times 1}$$

where **Γ**_*q*_ is the *q*-th integrated pavement plane.

The pavement plane is derived from Eq. (8). Although the homography is determined by the relationship between the image plane and the pavement plane, there are no enough constraints to solve the homography from two planes above. Hence, we propose a method to generate the homography from the perpendicular feet ${{\bf{M}}}_{q,j}^{{\rm{V}}}$ of the four laser points.

Considering the condition of the perpendicular feet on the integrated pavement plane^31^, the perpendicular foot satisfies

9 $${({{\boldsymbol{\Gamma }}}_{q})}^{{\rm{T}}}{{\bf{M}}}_{q,j}^{{\rm{V}}}=0.$$

We construct the vector consisting of the laser point and its perpendicular foot $({{\bf{M}}}_{q,j}^{{\rm{R}}}-{{\bf{M}}}_{q,j}^{{\rm{V}}})$. ${{\bf{M}}}_{q,k}^{{\rm{V}}}$ and ${{\bf{M}}}_{q,k-1}^{{\rm{V}}}$ are two points different from ${{\bf{M}}}_{q,j}^{{\rm{V}}}$ on the pavement plane. The vector $({{\bf{M}}}_{q,j}^{{\rm{R}}}-{{\bf{M}}}_{q,j}^{{\rm{V}}})$ is orthogonal to the vectors $({{\bf{M}}}_{q,k}^{{\rm{V}}}-{{\bf{M}}}_{q,k-1}^{{\rm{V}}})$ in the pavement plane. Thus,

10 $$({{\bf{M}}}_{q,j}^{{\rm{R}}}-{{\bf{M}}}_{q,j}^{{\rm{V}}})\bullet ({{\bf{M}}}_{q,k}^{{\rm{V}}}-{{\bf{M}}}_{q,k-1}^{{\rm{V}}})=0.$$

The perpendicular feet of the four laser points are solved by stacking Eqs (9) and (10). The homography H_*q*_ is then solved by^32^

11 $${{\rm{H}}}_{q}{{\bf{M}}}_{q,j}^{{\rm{V}}}\times {{\bf{m}}}_{q,j}^{{\rm{R}}}={\bf{0}}.$$

**Figure 4:**
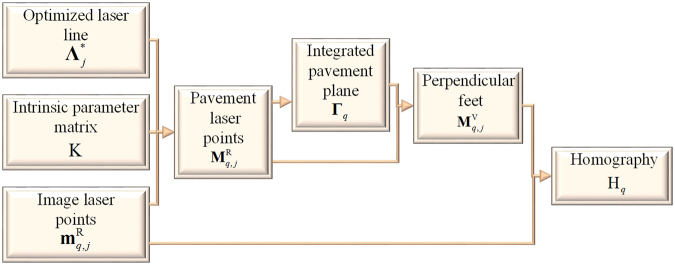
The active solution process of the homography on the basis of the four laser projection points.

The homography H_*q*_ is determined by the singular value decomposition (SVD) method^34^. The active solution process of the homography H_*q*_ that is generated from the four laser points is shown in Fig. 4.

## Results

**Figure 5:**
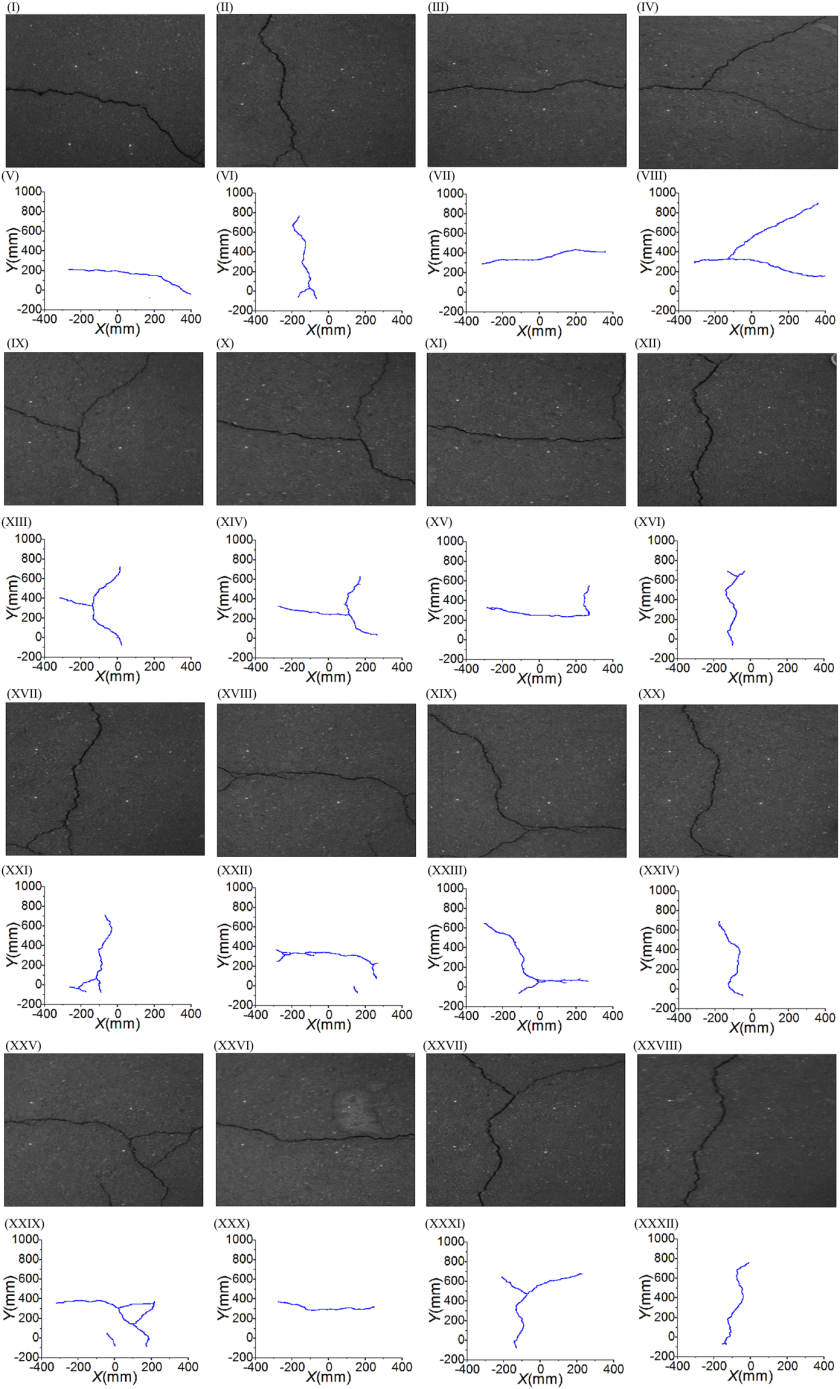
Recovery experiments of the pavement cracks that are achieved by the active solutions of the homographies. (**I**)–(**IV**), (**IX**)–(**XIl**), (**XVII**)–(**XX**), (**XXV**)–(**XXVIII**) are the eight images of the different pavement cracks. (**V**)–(**VIII**), (**XIII**)–(**XVI**), (**XXI**)–(**XXIV**), (**XXIX**)–(**XXXII**) are the recovery results of the different pavement cracks.

The experiments are performed by an Industrial Vision HT-U300C camera, which has the 2048 × 1536 image resolution and 3.0 megapixels. It is an industrial camera with the focus scope of 4 mm-12 mm and an aperture of F1.6. The laser line is generated from a Class IIIa Product SYD1230 laser projector. The output power of the line-laser projectors is 20 mW and the peak wavelength is 650 nm. The 2D target is a 150 mm × 150 mm board that is covered by the 10 mm × 10 mm rectangles. First, the camera is calibrated by the target board to obtain the internal and external parameters of the camera. The laser projectors provide four laser lines on the target and generate four laser points. The positions of the four laser lines are solved by the intersection laser points on the target. Then, the laser lines are projected to the pavement. The camera captures the images of the cracks and the laser projections. The homography is generated from the laser projections on the pavement. Finally, the pavement cracks are extracted in the image and transformed to the pavement plane by the active solution of the homography. The recovery results of the pavement cracks are shown in Fig. 5. Figure 5(a)–(d) and (i)–(l) are the pavement crack images. Figure 5(e–h) and (m–p) are the reconstructed pavement cracks. According to the active solution of the homography, the image coordinates of the pavement cracks are transformed to the real-dimension coordinates on the pavement plane.

**Figure 6:**
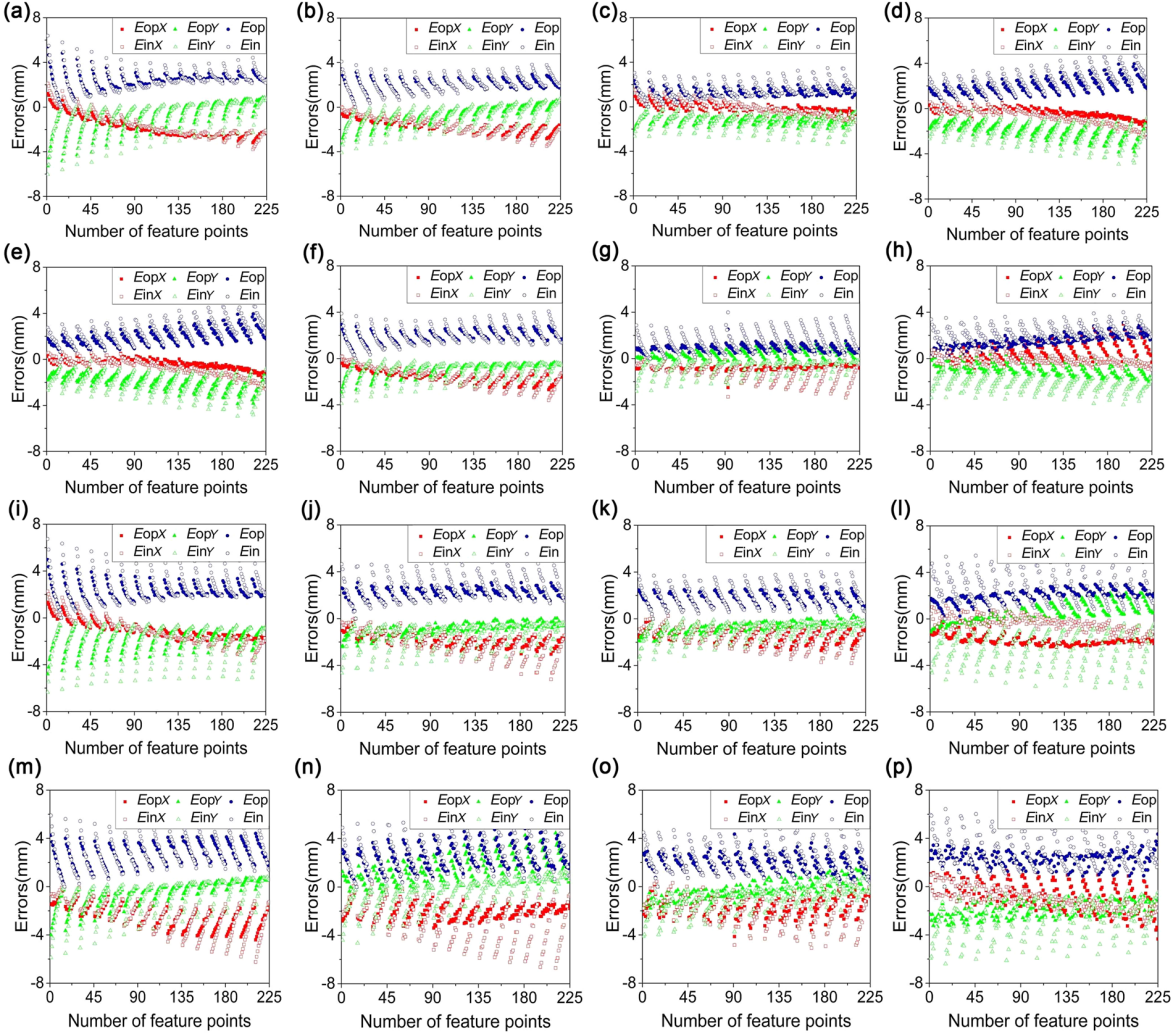
Recovery errors of the active solutions of the homographies in the verification experiments. The subscript “in” indicates the initialization method. The subscript “op” indicates the optimization method. The subscripts “*X*” and “*Y*” indicate the errors along the *X* direction or *Y* direction. E_in_ and E_op_ are the combined errors of the initialization method and the optimization method. MD indicates the measurement distance, mm. RA indicates the relative angle, °. (**a**) MD = 500, RA = 4. (**b**) MD = 600, RA = 4. (**c**) MD = 700, RA = 4. (**d**) MD = 800, RA = 4. (**e**) MD = 500, RA = 8. (**f**) MD = 600, RA = 8. (**g**) MD = 700, RA = 8. (**h**) MD = 800, RA = 8. (**i**) MD = 500, RA = 12. (**j**) MD = 600, RA = 12. (**k**) MD = 700, RA = 12. (**l**) MD = 800, RA = 12. (**m**) MD = 500, RA = 16. (**n**) MD = 600, RA = 16. (**o**) MD = 700, RA = 16. (**p**) MD = 800, RA = 16.

**Figure 7:**
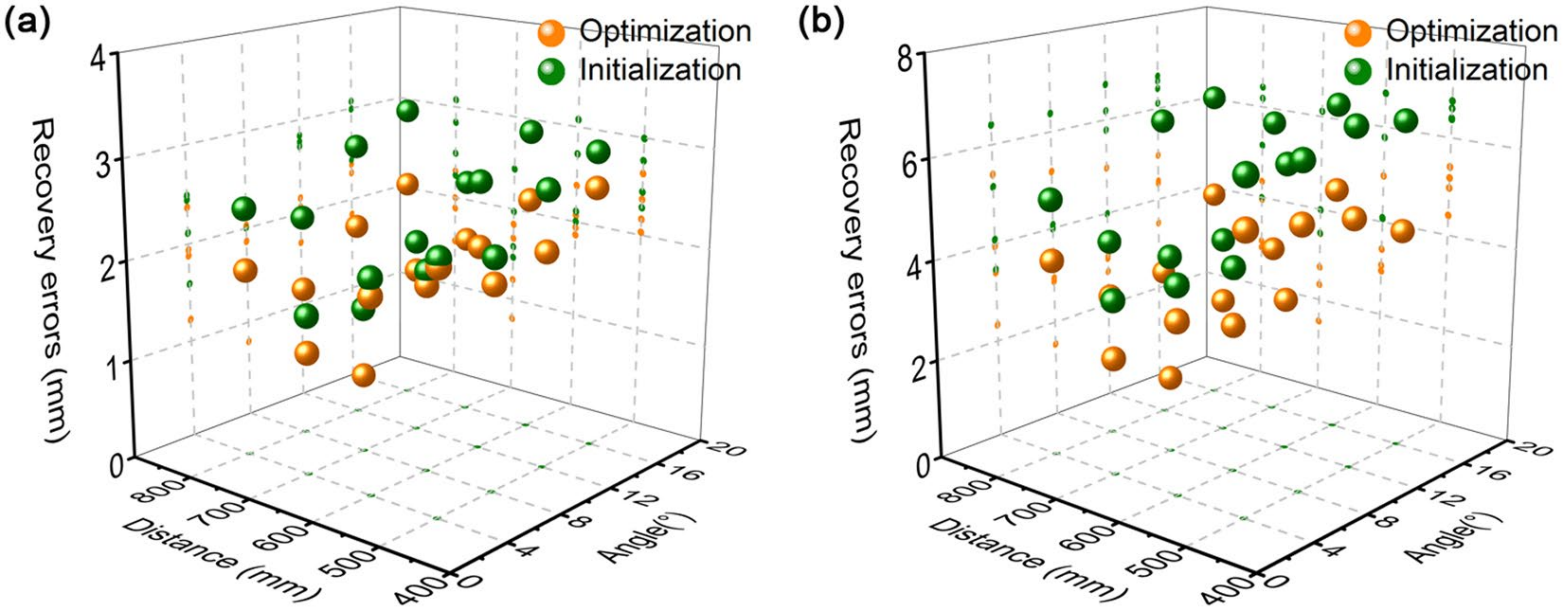
The statistical means and maximums of the recovery errors under the measurement distances of 500 mm, 600 mm, 700 mm, 800 mm, and the relative angles of 4°, 8°, 10°, 12°. (**a**) The statistical means of the optimization method and initialization method. (**b**) The statistical maximums of the optimization method and initialization method.

In order to evaluate the accuracy of the active solution method, the recovery errors of the homography are verified by experiments. The 225 corners of the checkerboard pattern on the target board are extracted as the feature points. The homography matrix is solved by the proposed method. The image coordinates of the 225 feature points are transformed to the real-dimension pavement coordinates by the homography. The recovery accuracy is evaluated by the differences between the reconstructed real-dimension coordinates and the real coordinates of feature points on the target. Two impact factors are considered in the experiments. One factor is the measurement distance between the target and the camera. The other factor is the relative angle between the optical axis of the camera and the normal vector of the target. In addition, the initialization method and the optimization method of the laser

line are used to calculate the recover errors to verify the accuracy of the homography. The experimental results are shown in Fig. 6. *E*_in*X*_ and *E*_in*Y*_ denote the *X*, *Y*-direction errors between the coordinates of the recovery feature points and the coordinates of the true feature points in the initialization method. *E*_op*X*_ and *E*_op*Y*_ denote the *X*, *Y*-direction errors between the coordinates of the recovery feature points and the coordinates of the true feature points in the optimization method. *E*_in_ and *E*_op_ represent the combined errors between the recovery feature points and the true feature points by the means of the initialization method and optimization method. Furthermore, Fig. 7 shows the statistical means and maximums of the errors between the recovery feature points and the real feature points under different experimental conditions. In Fig. 7(a), the golden balls and the green balls represent the means of the errors between the recovery feature points and the real feature points in the optimization method and initialization method, respectively. In Fig. 7(b), the golden balls and the green balls represent the maximums of the errors between the recovery feature points and the real feature points in the optimization method and initialization method, respectively.

In Fig. 7, when the relative angle between the normal vector of the target and the optical axis of the camera is 4° and the measurement distances between the target and the camera are 500 mm, 600 mm, 700 mm and 800 mm, the means of the recovery errors are 2.37 mm, 1.94 mm, 1.22 mm and 1.88 mm in the optimization method. The corresponding maximums of the errors are 5.41 mm, 3.41 mm, 2.34 mm and 4.00 mm. Moreover, for the initialization method, the means of the errors are 2.45 mm, 2.11 mm, 1.59 mm and 2.50 mm. the corresponding maximums are 6.39 mm, 4.09 mm, 3.48 mm and 5.16 mm. The recovery errors derived from the optimization method are smaller than the errors from the initialization method. It can be observed that the recovery errors decrease evidently when the measurement distance increases from 500 mm to 700 mm. However, when the measurement distance is up to 800 mm, the recovery errors become larger. Thus, in the test results under the relative angle of 4°, the recovery errors are smaller than others when the measurement distance is 700 mm. The test results correspond to Fig. 6(a–d). The recovery errors of Fig. 6(c) are more concentrative to zero than the errors of others.

The second group of tests is achieved by the relative angle of 8° and the measurement distances of 500 mm, 600 mm, 700 mm and 800 mm. The means of recovery errors are 2.03 mm, 1.86 mm, 0.79 mm and 1.51 mm in the optimization method in Fig. 7. The corresponding maximums are 5.18 mm, 2.94 mm, 1.52 mm and 2.87 mm in the optimization method. Furthermore, for the initialization method, the recovery errors are 2.29 mm, 2.00 mm, 1.47 mm and 2.25 mm. The related maximums are 6.37 mm, 4.07 mm, 3.98 mm and 4.00 mm. In this case the conclusion can be reached that when the measurement distance rises from 500 mm to 700 mm, the errors of recovery experiments decrease significantly. Then the recovery errors grow up on the condition that the measurement distance is 800 mm. Figure 6(e–h) relates to the group of experiments. The recovery errors of Fig. 6(g) are closer to zero than others. The tendencies of recovery errors of the optimization method and the initialization method are the same. In addition, the green balls are obviously higher than the golden balls in Fig. 7. So the recovery errors from the optimization method are smaller than the errors from the initialization method.

When the relative angle is 12° and the measurement distances are 500 mm, 600 mm, 700 mm and 800 mm, the means of recovery errors are 2.17 mm, 2.06 mm, 1.68 mm and 2.00 mm in the optimization method. The corresponding maximums are 4.98 mm, 3.07 mm, 2.72 mm and 3.02 mm. Besides, the means of the recovery errors are 2.77 mm, 2.72 mm, 1.98 mm and 2.83 mm in the initialization method. The corresponding maximums are 6.74 mm, 5.78 mm, 3.99 mm and 6.19 mm. The smallest errors in this test group are observed with respect to the measurement distance of 700 mm. The recovery errors in Fig. 6(k) are more approaching to zero than errors of others in Fig. 6(i–l). The green balls are higher than the golden balls. Therefore, the recovery errors derived from the optimization method are smaller than the errors from the initialization method.

The last group of experiments is performed by the relative angle of 16° and the measurement distances of 500 mm, 600 mm, 700 mm and 800 mm. The means of the recovery errors are 2.64 mm, 2.39 mm, 1.84 mm and 2.30 mm in the optimization method in Fig. 7. The corresponding maximums are 4.41 mm, 5.00 mm, 3.47 mm and 4.36 mm. Then, the means of the recovery errors are 3.00 mm, 3.08 mm, 2.44 mm and 3.08 mm and the corresponding maximums are 6.59 mm, 6.70 mm, 6.11 mm and 6.43 mm in the initialization method. The smaller errors are contributed when the relative angle is 12° and the measurement distance is 700 mm. The small errors from the optimization method are also observed in Fig. 7. The error reductions of the optimization method relative to the initialization method are described in Table 1, under the measurement distances of 500 mm, 600 mm, 700 mm, 800 mm, and the measurement angles of 4°, 8°, 12°, 16°. The average error reduction of the optimization method is 20.33%.

**Table 1:** The error reductions of the optimization method relative to the initialization method, under the measurement distances of 500 mm, 600 mm, 700 mm, 800 mm, and the measurement angles of 4°, 8°, 12°, 16°.

Distance (mm)	Angle (°)	Relative errors (mm)		Error reductions (mm)
		Initialization	Optimization	
500	4	2.45	2.37	0.08
	8	2.29	2.03	0.26
	12	2.77	2.17	0.60
	16	3.00	2.64	0.36
600	4	2.11	1.94	0.17
	8	2.00	1.86	0.14
	12	2.72	2.06	0.66
	16	3.08	2.39	0.69
700	4	1.59	1.22	0.37
	8	1.47	0.79	0.68
	12	1.98	1.68	0.30
	16	2.44	1.84	0.60
800	4	2.50	1.88	0.62
	8	2.25	1.51	0.74
	12	2.83	2.00	0.83
	16	3.08	2.30	0.78

## Discussion

In the test, the relative angle between the normal vector of the target and the optical axis of the camera increases from 4° to 16° with the interval of 4°. The means of the recovery errors are 1.85 mm, 1.55 mm, 1.98 mm and 2.29 mm in the optimization method and 2.16 mm, 2.00 mm, 2.57 mm and 2.90 mm in the initialization method. Hence, the recovery errors decrease when the relative angle increases from 4° to 8°. Recovery errors show a trend of steady growth when the relative angle increases from 8° to 16°. Moreover, when the angle is 12°, the errors are greater than 4° but less than 16°. Due to the virtual camera optical axis, it is impossible to obtain the true relative angle between the normal vector of the target and the optical axis of the camera. So the small relative angles generally contribute the better results than the large angles. Furthermore, the measurement distance between the camera and the measured object is also a factor that affects the errors of recovery experiments. The measurement distance increases from 500 mm to 800 mm, the means of the recovery errors are 2.31 mm, 2.06 mm, 1.38 mm and 1.92 mm in the optimization method and 2.62 mm, 2.48 mm, 1.87 mm and 2.66 mm in initialization method. So the errors of the recovery experiments are minimal when the distance is 700 mm. The errors of the recovery experiments obviously decrease when the distance increases from 500 mm to 700 mm. When the measurement distance is 800 mm, the recovery errors are larger than the errors at the distance of 700 mm, but slightly smaller than 600 mm. In summary, the experimental results show that the recovery values are closest to the real values when the measurement distance is 700 mm and the relative angle is 8°. The recovery errors of the optimization method are less than the errors of the initialization method in the verification experiments. Therefore, the optimization of the four laser lines reduces the experimental errors effectively. As the homography between the image plane of the camera and the base plane of the measured object plays an important role in various inspections, the active solution method of the homography for the pavement crack recovery with four laser lines can be widely popularized to the measurements of mechanical parts, electronic devices, architecture, etc.

## Summary

An active solution method of the homography is presented to recover the pavement cracks. The homography is generated from the pavement projections of four laser lines. The measurement distance between the camera and the target as well as the relative angle between the normal vector of the target and the optical axis of the camera are considered as the two impact factors on the recovery errors in the experiments. The global mean of the recovery errors of the initialization method is 2.41 mm and the global mean of the recovery errors of the optimization method is 1.91 mm. The experimental results show that the active solution method of the homography is a valid and accurate approach in the research field of the vision-based pavement measurement. Furthermore, for other vision-based inspections, it is also important to generate the homography from the dimension of the image plane to the dimension of the base plane. Therefore, the active solution method of the homography for the pavement crack recovery with four laser lines have the potentials to the measurements of mechanical parts, electronic devices, architecture, etc. In future work, the enhancement method to reduce the sunlight influence should be investigated for further applications.

### Data availability

The datasets generated during the current study are available from the corresponding author on reasonable request.
